# Dr. Byron C. Pennington

**Published:** 1905-02

**Authors:** 


Dr. Byron C. Pennington, of Atlantic City, N. J., died at his home January 1, 1905, aged 47 years. He had been in failing health for some time, having visited Bermuda in the early winter, hoping to stay the progress of a disease that was already manifesting a dangerous attitude. Dr. Pennington was elected one of the vice-presidents of the American Medical Association at its last annual meeting.

